# Supplementary material for: Single sperm karyotyping of testicular sperm in non-obstructive and obstructive azoospermia using next generation sequencing
Source: PLoS One. 2025 Dec 5;20(12):e0338222. doi: 10.1371/journal.pone.0338222 (PMC12680224; doi:10.1371/journal.pone.0338222)
Supplement: S1 Dataset — Karyotype analysis results for each sperm of control, BT, OA and NOA groups. WGA cut off <5ng/µl. (PDF) [file pone.0338222.s001.pdf]

S1 Dataset. Karyotype analysis results for each sperm

Group	Patient #	Sample	QC	Karyotyping result	Conclusion	WGA amount (ng/ul)	Total reads
Control	1	1	Pass	23,X	Normal	32.9	333780
Control	1	2	Pass	23,X	Normal	36.2	403779
Control	1	3	Pass	23,Y	Normal	32.5	275454
Control	1	4	Pass	23,Y	Normal	34.8	343541
Control	1	5	Pass	23,X	Normal	32.4	274204
Control	1	6	Pass	23,X	Normal	31.8	309171
Control	1	7	Pass	23,X	Normal	30.5	238004
Control	1	8	Pass	23,X	Normal	29.6	244894
Control	1	9	Pass	23,Y	Normal	28.3	233023
Control	1	10	Pass	23,X	Normal	32.4	255702
Control	2	1	Fail	Too many noise	NA	19	178930
Control	2	2	Pass	23,Y	Normal	34.6	286702
Control	2	3	Pass	23,Y	Normal	29.4	246303
Control	2	4	Pass	23,X	Normal	46.2	357140
Control	2	5	Pass	23,Y	Normal	34.8	288102
Control	2	6	Pass	23,X	Normal	38.1	310385
Control	2	7	Fail	Too many noise	NA	17.7	134994
Control	2	8	Pass	23,Y	Normal	44.1	301712
Control	2	9	Pass	23,X	Normal	36.1	304517
Control	2	10	Fail	Amplification failure	NA	2.5	11830
Control	3	1	Pass	23,X	Normal	33.2	235255
Control	3	2	Pass	23,Y	Normal	35.3	309305
Control	3	3	Fail	Amplification failure	NA	4.8	39437
Control	3	4	Pass	23,Y	Normal	35.9	282932
Control	3	5	Pass	23,X	Normal	39.3	349307
Control	3	6	Pass	23,X	Normal	29.7	254028
Control	3	7	Pass	23,X	Normal	37.9	321719
Control	3	8	Pass	23,X	Normal	19.6	157883
Control	3	9	Pass	23,X	Normal	35	324538
Control	3	10	Pass	23,X	Normal	31.2	390050
Control	4	1	Pass	23,Y	Normal	32.1	232475
Control	4	2	Pass	23,Y	Normal	41.1	379364
Control	4	3	Pass	23,Y	Normal	35.6	281658
Control	4	4	Pass	23,X	Normal	43.9	360616
Control	4	5	Pass	23,X	Normal	31	243236
Control	4	6	Pass	23,X	Normal	39.9	321856
Control	4	7	Pass	23,X	Normal	34.6	292571
Control	4	8	Pass	23,X	Normal	41.5	315079
Control	4	9	Pass	23,Y	Normal	31.5	255216
Control	4	10	Pass	23,Y	Normal	30.3	238007

Group	Patient #	Sample	QC	Karyotyping result	Conclusion	WGA amount (ng/ul)	Total reads
BT	5	1	Pass	23,X	Normal	17.4	182383
BT	5	2	Pass	23,Y	Normal	45.6	389147
BT	5	3	Pass	22,(-X or Y)	Aberrant	46	351880
BT	5	4	Pass	23,X	Normal	46.1	383057
BT	5	5	Pass	23,X	Normal	17	123358
BT	5	6	Pass	23,X	Normal	39.1	234921
BT	5	7	Pass	23,Y	Normal	37.7	276608
BT	5	8	Pass	23,Y	Normal	39.9	316254
BT	5	9	Fail	Amplification failure	NA	2.3	5388
BT	5	10	Pass	22,X,-14	Aberrant (Unbalanced)	36.1	282258
BT	6	1	Fail	Amplification failure	NA	4.9	22787
BT	6	2	Pass	23,X,der(22)t(19;22)(q13.4;13.2)	Aberrant (Unbalanced)	42	321552
BT	6	3	Pass	23,X,der(19)t(19;22)(q13.4;13.2)	Aberrant (Unbalanced)	34.2	253943
BT	6	4	Pass	23,X	Normal	35.7	233808
BT	6	5	Pass	23,X	Normal	32	226394
BT	6	6	Pass	23,X	Normal	37	244714
BT	6	7	Pass	23,X	Normal	34.8	254555
BT	6	8	Pass	23,X,der(19)t(19;22)(q13.4;13.2)	Aberrant (Unbalanced)	39.9	254598
BT	6	9	Pass	23,Y	Normal	22.2	139410
BT	6	10	Pass	23,X,der(22)t(19;22)(q13.4;13.2)	Aberrant (Unbalanced)	33.1	221155
BT	7	1	Pass	23,X,der(6)t(6;16)(q11;p11.2))	Aberrant (Unbalanced)	31.8	291207
BT	7	2	Pass	23,X	Normal	34.9	282014
BT	7	3	Pass	23,X,-6,+der(16)t(6;16)(q11;p11.2)	Aberrant (Unbalanced)	31.9	269739
BT	7	4	Pass	23,X	Normal	36.4	312734
BT	7	5	Pass	23,X,-6,+der(16)t(6;16)(q11;p11.2)	Aberrant (Unbalanced)	34.5	294543
BT	7	6	Pass	23,X,+der(6)t(6;16)(q11;p11.2),-16	Aberrant (Unbalanced)	37.2	345463
BT	7	7	Pass	23,X,der(16)t(6;16)(q11;p11.2))	Aberrant (Unbalanced)	35.4	349614
BT	7	8	Pass	23,Y	Normal	31.4	303682
BT	7	9	Pass	23,X	Normal	37.9	436059
BT	7	10	Pass	23,X	Normal	28.4	198667

Group	Patient #	Sample	QC	Karyotyping result	Conclusion	WGA amount (ng/ul)	Total reads
OA	8	1	Pass	23,Y	Normal	45.6	466721
OA	8	2	Pass	23,Y	Normal	38.2	435198
OA	8	3	Pass	23,X	Normal	42.9	417865
OA	8	4	Fail	Amplification failure	NA	38.6	344538
OA	8	5	Pass	23,X	Normal	43.3	457802
OA	8	6	Pass	23,X	Normal	42.2	358263
OA	8	7	Pass	23,X	Normal	41	422973
OA	8	8	Fail	Amplification failure	NA	2.4	16175
OA	8	9	Fail	Amplification failure	NA	2	8811
OA	8	10	Fail	Amplification failure	NA	2.2	11972
OA	9	1	Pass	23,Y	Normal	29.8	396746
OA	9	2	Pass	23,Y	Normal	25.7	331736
OA	9	3	Pass	23,X	Normal	26.4	403407
OA	9	4	Pass	23,Y	Normal	31.8	408050
OA	9	5	Pass	23,Y	Normal	30.1	336179
OA	9	6	Pass	23,Y	Normal	24.2	297385
OA	9	7	Pass	23,Y	Normal	22.1	285781
OA	9	8	Pass	23,Y	Normal	21.7	254523
OA	9	9	Pass	23,Y	Normal	25.7	356680
OA	9	10	Pass	23,X	Normal	29	293567
OA	10	1	Fail	Amplification failure	NA	3.6	29772
OA	10	2	Pass	23,X	Normal	26.3	365217
OA	10	3	Pass	23,Y	Normal	25	322738
OA	10	4	Pass	23,X	Normal	26.5	359001
OA	10	5	Fail	Too many noise	NA	7.5	97156
OA	10	6	Pass	23,Y	Normal	19	253893
OA	10	7	Pass	23,X	Normal	23.8	251265
OA	10	8	Fail	Amplification failure	NA	1.8	5994
OA	10	9	Fail	Amplification failure	NA	1.4	5716
OA	10	10	Pass	23,Y	Normal	19.5	217235
OA	11	1	Pass	23,X	Normal	30.6	412539
OA	11	2	Fail	Uneven amplification	NA	29.5	429543
OA	11	3	Pass	23,Y	Normal	25.9	377588
OA	11	4	Pass	23,X	Normal	32.8	433936
OA	11	5	Pass	23,X	Normal	36.4	425871
OA	11	6	Pass	23,Y	Normal	35.4	448573
OA	11	7	Pass	23,Y	Normal	35.2	439149
OA	11	8	Pass	23,X	Normal	34.6	445213
OA	11	9	Pass	23,X	Normal	13.2	181872
OA	11	10	Pass	23,X	Normal	24.1	283141
OA	12	1	Pass	23,Y	Normal	43.1	421607
OA	12	2	Pass	23,X	Normal	37.4	341981
OA	12	3	Pass	23,Y	Normal	29.3	255424
OA	12	4	Pass	23,Y	Normal	37.8	276593
OA	12	5	Pass	23,X	Normal	35.3	259720
OA	12	6	Pass	23,X	Normal	24.4	194432
OA	12	7	Fail	Amplification failure	NA	3.7	24799
OA	12	8	Pass	23,X	Normal	35.5	340793
OA	12	9	Pass	23,Y	Normal	22.5	171482
OA	12	10	Pass	23,X	Normal	37.3	368784

Group	Patient #	Sample	QC	Karyotyping result	Conclusion	WGA amount (ng/ul)	Total reads
NOA	13	1	Pass	23,Y	Normal	27.5	359962
NOA	13	2	Pass	23,Y	Normal	30.6	425955
NOA	13	3	Pass	23,X	Normal	29.3	464984
NOA	13	4	Pass	23,X	Normal	28.6	392592
NOA	13	5	Pass	23,X	Normal	28.7	398554
NOA	13	6	Pass	23,X	Normal	26.6	337483
NOA	13	7	Fail	Amplification failure	NA	1.9	7386
NOA	13	8	Pass	23,X	Normal	22.3	290228
NOA	13	9	Pass	23,Y, dup(15)(pter→q11.2)	Aberrant	25.6	374374
NOA	13	10	Pass	23,Y	Normal	28.2	332392
NOA	14	1	Fail	Amplification failure	NA	27.4	897456
NOA	14	2	Pass	23,X	Normal	41.2	487366
NOA	14	3	Fail	Amplification failure	NA	5.2	72982
NOA	14	4	Pass	23,X	Normal	42.8	491389
NOA	14	5	Pass	23,X	Normal	34.8	389999
NOA	14	6	Pass	23,X	Normal	38.9	401857
NOA	14	7	Pass	23,X	Normal	38.4	425656
NOA	14	8	Pass	23,X	Normal	39.1	439610
NOA	14	9	Fail	Uneven amplification	NA	46	721395
NOA	14	10	Pass	23,Y	Normal	27.4	131048
NOA	15	1	Pass	23,Y	Normal	39.4	286789
NOA	15	2	Pass	24,Y,+7	Aberrant	45.2	414348
NOA	15	3	Pass	23,X	Normal	38.3	319710
NOA	15	4	Pass	24,Y,+7	Aberrant	36.7	333641
NOA	15	5	Fail	Too many noise	Aberrant	31	211149
NOA	15	6	Pass	23,Y	Normal	32.3	196203
NOA	15	7	Pass	22,Y,-14	Aberrant	33.2	257842
NOA	15	8	Pass	20,-1,-5	Aberrant	24.7	186537
NOA	15	9	Pass	23,X	Normal	33.6	270791
NOA	15	10	Pass	24,Y,+8,del(1)(q42.2→qter)	Aberrant	32.5	217921
NOA	16	1	Pass	23,X	Normal	39.7	303567
NOA	16	2	Pass	23,X	Normal	35.9	232470
NOA	16	3	Fail	Too many noise	NA	25.9	194588
NOA	16	4	Pass	23,X	Normal	30.1	220015
NOA	16	5	Pass	23,X	Normal	30.9	238676
NOA	16	6	Pass	23,X,del(11)(pterp→11.12)	Aberrant	27	215010
NOA	16	7	Pass	23,Y	Normal	36	272085
NOA	16	8	Pass	23,X	Normal	35.8	287211
NOA	16	9	Pass	23,Y	Normal	34.9	257772
NOA	16	10	Fail	Too many noise	NA	25.7	190032
NOA	17	1	Pass	23,Y	Normal	30	503373
NOA	17	2	Pass	23,Y	Normal	32	461487
NOA	17	3	Pass	23,X	Normal	33.5	510571
NOA	17	4	Pass	23,X	Normal	30.5	430511
NOA	17	5	Pass	23,X	Normal	35	444356
NOA	17	6	Fail	Too many noise	NA	29	330280
NOA	17	7	Pass	23,Y	Normal	35.7	461605
NOA	17	8	Pass	23,X	Normal	35	413665
NOA	17	9	Fail	Amplification failure	NA	2.1	5875
NOA	17	10	Pass	23,Y	Normal	37.2	440320
