# Supplementary material for: Single sperm karyotyping of testicular sperm in non-obstructive and obstructive azoospermia using next generation sequencing
Source: PLoS One. 2025 Dec 5;20(12):e0338222. doi: 10.1371/journal.pone.0338222 (PMC12680224; doi:10.1371/journal.pone.0338222)
Supplement: S2 Dataset — Individual karyotyping success rates (number of karyotyping-successful sperm/ number of analyzed sperm) did not significantly correlate with motile sperm count at TESE (R = 0.464, P = 0.177). Individual aberrant sperm detection rates (number of aberrant sperm/ number of karyotyping-successful sperm) did not correlate with motile sperm count at TESE (R = −0.149, P = 0.681). (PDF) [file pone.0338222.s002.pdf]

S2 Dataset.

Individual karyotyping success rates

| Patient # | Group | motile sperm count at TESE | number of karyotyping-successful sperm | number of analyzed sperm | karyotyping success rate % |
|---|---|---|---|---|---|
| 8 | OA | 1600 | 6 | 10 | 60 |
| 9 | OA | 46800 | 10 | 10 | 100 |
| 10 | OA | 1600 | 6 | 10 | 60 |
| 11 | OA | 1600 | 9 | 10 | 90 |
| 12 | OA | 3000 | 9 | 10 | 90 |
| 13 | NOA | 200 | 9 | 10 | 90 |
| 14 | NOA | 1680 | 7 | 10 | 70 |
| 15 | NOA | 1000 | 9 | 10 | 90 |
| 16 | NOA | 17600 | 8 | 10 | 80 |
| 17 | NOA | 3200 | 8 | 10 | 80 |

Individual aberrant sperm detection rates

| Patient # | Group | motile sperm count at TESE | number of aberrant sperm | number of karyotyping-successful sperm | aberrant sperm detection rates % |
|---|---|---|---|---|---|
| 8 | OA | 1600 | 0 | 6 | 0% |
| 9 | OA | 46800 | 0 | 10 | 0% |
| 10 | OA | 1600 | 0 | 6 | 0% |
| 11 | OA | 1600 | 0 | 9 | 0% |
| 12 | OA | 3000 | 0 | 9 | 0% |
| 13 | NOA | 200 | 1 | 9 | 11% |
| 14 | NOA | 1680 | 0 | 7 | 0% |
| 15 | NOA | 1000 | 5 | 9 | 56% |
| 16 | NOA | 17600 | 1 | 8 | 13% |
| 17 | NOA | 3200 | 0 | 8 | 0% |
