# Supplementary material for: Single sperm karyotyping of testicular sperm in non-obstructive and obstructive azoospermia using next generation sequencing
Source: PLoS One. 2025 Dec 5;20(12):e0338222. doi: 10.1371/journal.pone.0338222 (PMC12680224; doi:10.1371/journal.pone.0338222)
Supplement: Attachment — Submitted filename: Response_to_Reviewers_letter.docx [file pone.0338222.s003.docx]

June 30, 2025

Dear Editor

Thank you for allowing us to resubmit our manuscript as a new submission (Case Number: 09070569). We would like to explain the points raised by staff editor Dr. Jianhong Zhou in our previous decision letter (PONE-D-25-29334).

Dr. Jianhong Zhou's comments are as follows.

**Based on our evaluation of your manuscript and the information you provided, we do not feel that your submission meets our ethical requirements for human subjects research submissions. PLOS ONE requires that research meets all applicable standards for the ethics of experimentation and research integrity (http://journals.plos.org/plosone/s/human-subjects-research). We reserve the right to reject any submission that does not meet our internal ethical standards, which in some cases are more stringent than local ethical standards.**

Our explanation regarding the above comment is as follows.

We would like to let you know we mistakenly sent inappropriate ethical documents in the previous submission (PONE-D-25-29334). The documents sent were unfortunately the approved research protocols, not approval documents. We apologize for submitting the incorrect documents in the previous submission. As a result, you had felt that our previous submission did not meet your ethical requirements. Because our university keeps a solid ethical committee, this was the first time to have a rejection decision by ethical documents. We believe if you take appropriate documents, you may feel our submission does meet your ethical requirement.

In a new submission (this submission) we have submitted appropriate documents. The submitted files are appropriate documents for ethical approval in our university. We would be most grateful if you kindly check these correct files and proceed with further review steps.

Thank you for your kind consideration.

Best regards,

Tomonari Hayama, M.D.,Ph.D.

Department of GYN, Yokohama City University Medical Center

4-57 Urafunacho, Minami-ku, Yokohama City, Kanagawa Prefecture, Japan.

Email: tommy_h@yokohama-cu.ac.jp
