# Supplementary material for: Single sperm karyotyping of testicular sperm in non-obstructive and obstructive azoospermia using next generation sequencing
Source: PLoS One. 2025 Dec 5;20(12):e0338222. doi: 10.1371/journal.pone.0338222 (PMC12680224; doi:10.1371/journal.pone.0338222)
Supplement: Attachment — Submitted filename: Response to Reviewers.docx [file pone.0338222.s004.docx]

[23. Oktober 2025]

Emily Chenette

Editor-in-Chief

PLOS One

Dear Editor and Reviewer:

We wish to submit the revised manuscript titled “Single sperm karyotyping of testicular sperm in non-obstructive and obstructive azoospermia using next generation sequencing.” The manuscript ID is PONE-D-25-35308.

We thank you and the reviewer for the thoughtful suggestions and insights. The manuscript has benefited from these insightful suggestions. I look forward to working with you and the reviewers to move this manuscript closer to publication in PLOS One.

The manuscript has been rechecked and the necessary changes have been made in accordance with the reviewers’ suggestions. The responses to all comments have been prepared and attached herewith. Simple corrections to English words and rearrangements of sentence order in the manuscript are marked in yellow. Portions where content has been changed in the manuscript are marked in light blue.

**Additional Editor Comments:**

The submission was evaluated by a cytogenetics expert who provided a detailed assessment, which you will find below. Given the significant weaknesses in the presentation of the data and their excessive interpretation in some cases, a “major revision” is recommended, while taking into account that the technology developed may be of some interest. Please carefully follow the reviewer's comments. Some sections could be condensed to avoid unnecessary repetition. In addition, the discussion should focus on your own data.

→We thank the editor for the valuable advice. Accordingly, we have removed unnecessary repetition (Line375-384) and we have focused on the interpretation of the results (Line385-405). We have also made revisions to the Figures (Fig 1-8) and S1 dataset.

**Review Comments to the Author**

Reviewer #1: I had the opportunity to review the paper entitled “Single sperm karyotyping of testicular sperm of non-obstructive and obstructive azoospermia using next generation sequencing” by Tomonari Hayama et al. The study aims to evaluate the incidence of chromosomal abnormalities in sperm from patients with non-obstructive azoospermia, considering previous studies that reported an increased incidence of such abnormalities. Although the results are not entirely novel, the methodology proposed here—based on approaches commonly used for PGT-A analysis—is of interest. However, the paper requires substantial revision before it can be considered for publication in PLOS ONE, particularly the discussion section, as explained below. In addition, there are numerous typographical errors, inappropriate sentences, and cytogenetic inaccuracies that must be corrected.

→Thank you for valuing our analysis method. Based on your comments and after consulting with a cytogeneticist, we have substantially revised the discussion section and figures. We have shared your comments and have asked an English editing service to re-edit the manuscript.

We hope that we have met your expectations.

Major remarks:

1- The discussion section, in its current form, is not suitable and needs to be revised and considerably reduced.

- the authors should provide a clear explanation of the reported 20% failure rate, and discuss the protocol in more detail, particularly its feasibility and reproducibility for other teams attempting to replicate this strategy.

→We thank the Reviewer for their kind suggestion. Accordingly, compared to a previous sperm NGS report, our method improves the karyotype acquisition rate by isolating sperm through micromanipulation. However, we believe that the 20% failure rate may be due to natural DNA degradation or fragmentation during sperm DNA preparation. We added an explanation in lines 396-405.

- the results should be discussed separately for each group in light of previous studies, with particular attention to the NOA group, where high variability has been observed. This discussion should also be connected to the high aneuploidy rate reported in oocytes.

→We thank the Reviewer for their valuable comment. We have added a discussion on the results for each group. In the control and the OA group, no aberrant sperm were observed. This is thought to be because the spermatogenic function of the testes is maintained in these two groups (Lines 416-420). In contrast, in the NOA group, there were patients who had azoospermia due to apoptosis caused by abnormal meiosis, which is thought to be why multiple aberrant sperm were observed (Lines 421-424). Additionally, we have focused on the discussion of the NOA group and revised the previous description (Lines 425-434).

- it is unnecessary to repeat large portions of the introduction at the beginning of the discussion. This section should instead focus on the interpretation of the results.

→We thank the Reviewer for their valuable advice. We have removed unnecessary repetition and briefly summarized the advantages of our method compared to previous studies (Lines 375-384). We have focused on the interpretation of the results and described that we were able to detect chromosomal abnormalities that could not be captured by conventional FISH methods. (Lines 385-395).

- please clarify why it could be relevant to propose PGT-A for couples similar to patient 15. In this specific case, was a sperm FISH analysis performed for chromosome 7?

→We thank the Reviewer for their kind suggestion. An explanation of TESE-ICSI has been added to the Introduction (Lines 110-119). In TESE-ICSI, it is necessary to select morphologically normal mature sperm suitable for ICSI from a limited number of sperm. In Patient #15, even with microscopic selection, multiple sperm exhibited chromosomal abnormalities. In such patients, a possibility that sperm with chromosomal abnormalities were actually injected into the egg during the TESE-ICSI exists, suggesting a high risk of chromosomal abnormalities in embryos attributable to the sperm. Therefore, proposing PGT-A to couples similar to patient #15 is one option (Lines 435-447). Since two sperm with chromosome 7 disomy were detected in patient #15, performing FISH for chromosome 7, as the Reviewer suggested, would be beneficial. However, in this analysis, only morphologically normal sperm selected microscopically were analyzed to reflect the karyotypes of sperm used clinically. Consequently, the number of analyzable sperm was very low, which made performing sperm FISH analysis for chromosome 7 unachievable. Furthermore, there were no samples available for FISH analysis after the NGS results were confirmed because we were using samples that were discarded after clinical use. Therefore, FISH analysis could not be performed.

- Finally, the discussion should focus more closely on the study results, avoiding excessive speculation. In several places, sentences extend beyond the scope of the findings and should be revised.

→We thank the Reviewer for their valuable feedback. We have revised the sections that appeared to contain excessive speculation. The revised sections are as follows:；

We rewrote sentence ‘single sperm sequencing may be useful for selecting patients who truly require PGT-A’ as sentence ‘single sperm sequencing may help identify patients with NOA most likely to benefit from PGT-A’ (Lines 46-47).

We rewrote sentence ‘Even with sperm selection for clinical use, NGS karyotyping still reveals chromosomal abnormalities that could not be previously detected.’ as sentence ‘We analyzed all chromosomes, therefore, in our cohort, we were able to detect additional abnormalities not captured by conventional FISH’ (Lines 388-389).

We rewrote sentence ‘provides more accurate information to determine whether patients should undergo PGT-A.’ as sentence ‘This may provide complementary information when considering whether PGT-A is appropriate.’ (Lines 446-447).

We rewrote sentence ‘it would be beneficial for patients to determine the level of necessity for PGT-A by sperm karyotype analysis, which is not invasive to embryos’ as sentence ‘when considering whether PGT-A is appropriate, it may provide additional insights without invasiveness to embryos’ (Lines 461-462).

We rewrote sentence ‘As the sequencing results have a higher resolution than those of previous conventional FISH methods, the application of single sperm sequencing may be useful in selecting patients who require PGT-A’ as sentence ‘Although validation using larger per-patient datasets is necessary, incorporating this assay may help identify patients with NOA most likely to benefit from PGT-A’ (Lines 468-470).

2- There are several cytogenetic mistakes and misinterpretations that significantly compromise the accuracy of the results. As a cytogeneticist, it is evident that the manuscript has not been critically reviewed by a specialist in this field. Below, I summarize the most important issues; however, I strongly recommend that the authors consult with an expert in cytogenetics to ensure accuracy and consistency throughout the paper.

Line 89: The authors refer to “an antibody,” but this is incorrect. It should be described as a specific DNA probe labeled with a fluorochrome.

→We thank the Reviewer for pointing this out. We have made the necessary correction. Additionally, we have revised the preceding and following sentences to make them more concise (Lines 82-87).

Line 231: The karyotypes are not reported according to ISCN nomenclature (e.g., “46XY” should be “46,XY”). In addition, the complete karyotype formula should be provided for the BT group, particularly for patients with reciprocal translocations, as this is essential for proper interpretation of the results.

→We apologize for the inaccurate description of the karyotype. The karyotypes have been entirely revised according to ISCN nomenclature. Additionally, the complete karyotypes have been provided for BT group patients. For example, we have rewritten the karyotype of patient #5 as ‘45,XY, der(13;14)(q10;q10)’ (Lines 241-242).

When describing the absence or duplication of a whole chromosome, the appropriate terms are nullisomy and disomy, respectively, and should be used consistently throughout the manuscript.

The section Sperm karyotyping of the BT group requires a thorough revision according to both translocation segregation patterns and ISCN nomenclature. For example, in patient 6, sample 3, the karyotype should be written as 23,X,+der(19)t(19;22),-19. This clarification is necessary for accurate interpretation. For the same patient, the reported karyotype 23,X,del(22) is questionable. It may in fact correspond to 23,X,+der(22)t(19;22),-22. The authors should carefully review this point.

→We apologize for the inconsistent description of the sperm karyotype. We have verified with a genetic expert, the translocation segregation patterns for the BT group (Lines 278-286, Fig 2 and Fig 4-6) and we have revised the sperm karyotype description (Lines 288-317, Lines 320-333, Table 4, Fig 4-6 and S1 Dataset). Regarding the sperm karyotype previously described as del(22), we considered that a translocation dup(19)(q13.43→qter), which is below the detection limit of our protocol, was not detected and the actual sperm karyotype was determined to be 23,X,der(22)t(19;22)(q13.4;13.2) (Lines 322-326). In addition, the karyotype description for the NOA group and Table5 have been revised to conform to ISCN nomenclature, in line with the description for the BT group (Lines 352-356, Fig 5 and Table 5). As a result, the terms nullisomy and disomy were not used. No sex chromosomes were detected in Sample 8 of Patient #15 in the NOA group. Because the ISCN does not define how to describe a karyotype when no sex chromosomes are present, in consultation with a cytogeneticist, the karyotype of Sample 8 was described as "20,-1,-5."

The manuscript contains numerous typographical errors and poorly constructed sentences. A thorough language revision is strongly recommended.

→We apologize for the errors in the manuscript. Although we used an English proofreading service prior to our initial submission, we have shared the Reviewer’s feedback and have requested a re-proofreading.

Line 369: Please clarify the mention of “chr 23.” This terminology is incorrect and should be revised.

→We apologize for the typographical error. We have corrected it.

Line 387: The phrase “zygote stage of meiosis” is inappropriate and scientifically incorrect. Please rephrase this sentence to accurately reflect the intended meaning.

→We thank the Reviewer for pointing this out. The mentioned phrase has been removed during current revision.

We hope our responses adequately address the concerns raised. We have carefully revised our manuscript to improve its quality and clarity. We appreciate the Editor’s and Reviewers' constructive feedback, and we hope that the revisions made will meet their approval. Once again, we thank the Editor and Reviewers very much for their comments and suggestions.

Thank you for your consideration. We look forward to hearing from you.

Best,

Tomonari Hayama, M.D.,Ph.D.

Department of GYN, Yokohama City University Medical Center

4-57 Urafunacho, Minami-ku, Yokohama City, Kanagawa Prefecture, Japan.

[Phone number] +81452615656

[Email] tommy_h@yokohama-cu.ac.jp
