# Supplementary material for: Single sperm karyotyping of testicular sperm in non-obstructive and obstructive azoospermia using next generation sequencing
Source: PLoS One. 2025 Dec 5;20(12):e0338222. doi: 10.1371/journal.pone.0338222 (PMC12680224; doi:10.1371/journal.pone.0338222)
Supplement: Attachment — Submitted filename: Nov18_Response to Reviewers.docx [file pone.0338222.s005.docx]

[November 18. 2025]

Emily Chenette

Editor-in-Chief

PLOS One

Dear Editor and Reviewer:

We are pleased to re-submit the manuscript titled “Single sperm karyotyping of testicular sperm in non-obstructive and obstructive azoospermia using next generation sequencing.” The manuscript ID is PONE-D-25-35308R1.

We thank you and the reviewer for your positive assessment of the revisions. We are grateful that you and the reviewers have helped us move this manuscript closer to publication.

The manuscript has been rechecked and the necessary changes have been made in accordance with the reviewers’ suggestions. The responses to all comments have been prepared and attached herewith. Simple corrections to English words and rearrangements of sentence order in the manuscript are marked in yellow. Portions where content has been changed in the manuscript are marked in light blue.

Additional Editor Comments:

After this first round of revisions, the manuscript has been significantly improved. However, there are still a few additional points that could be addressed to make it even better.

I am therefore returning it to you so that you make a few more minor changes. Please note that this will be the final revision phase offered by the editorial office, so try to cover all the points raised by the reviewer.

→We appreciate your advice. We have addressed each of the points raised by the reviewers.

We have also corrected typographical errors in the CNV descriptions within Table 5 and Figure 8D, and the updated files (Nov18‗Revised Fig8.TIF) have been uploaded accordingly. We sincerely apologize for any inconvenience this may have caused and kindly request your confirmation.

Reviewer #1: I have had the opportunity to review for the second time the manuscript entitled “Single sperm karyotyping of testicular sperm in non-obstructive and obstructive azoospermia using next generation sequencing” submitted to “Plos One”. The paper has been clearly improved and seems to be suitable for publication after few improvements, especially concerning the redundancy.

→We thank the Reviewer for evaluating the improvements. We have made revisions based on your comments. We hope that we have met your expectations.

One point is worth discussing. You clearly confirm a high rate of chromosomal abnormalities in the sperm of patients with NOA. However, before proposing single sperm sequencing prior to PGT-A, you need to consider several points:

- The cost: on average, 5 to 7 blastocysts can be expected per ICSI. Compared to the analysis of 10 spermatozoa, PGT-A therefore seems less expensive.

- The effectiveness of PGT-A in detecting oocyte aneuploidy, which is impossible to achieve with a sperm analysis.

- The risk associated with biopsy and embryo freezing/thawing. This risk appears to be very low today.

Therefore, in my opinion, you are proposing a very interesting new technique, but one that is difficult to implement in medical practice because it is less relevant than PGT-A analysis and non-invasive PGT-A using culture media.

→We thank the Reviewer for their valuable feedback. As you pointed out, we believe that there is little cost benefit to single sperm sequencing at this time. We have removed the reference to cost (Lines 415-416).

As you mentioned, the risks associated with embryo biopsy and freezing and thawing are thought to be low, but the "long-term" prognosis after adulthood is still unknown. For this reason, some patients are hesitant to undergo PGT-A, wanting to avoid embryonic damage as much as possible. Furthermore, the fertilization rate with TESE-ICSI is lower than that of the general population, and depending on the number of oocytes collected, there are cases where only one or two embryos can be biopsied. In such cases, the risk of embryo invasiveness by PGT-A is undesirable.

Given this background, we believe that single sperm sequencing, by which we can obtain accurate sperm chromosomal information without embryo invasiveness, may be able to screen patients with multiple number of sperm chromosomal abnormalities, such as Patient #15, and may provide information that will be useful in determining the need for PGT-A (Lines 421-426).

On the other hand, we do not believe that single sperm sequencing, which can only detect chromosomal abnormalities of male origin, can be a more effective method than PGT-A, which can also detect aneuploidy of female origin (Lines 426-429). Although the target population will be limited, we hope to contribute to the development of this field by performing single sperm sequencing and accumulating sperm karyotype data in the future.

Regarding the structure of the text, here are a few comments:

The introduction could be shortened, and some elements could be deleted.

There are errors, redundancies, and repetitions; see some examples according to lines number in the highlighted version.

→We thank the Reviewer for your constructive feedback. We have shortened the Introduction by removing the redundant statement that was previously written on line 110. We have responded to each of the examples you provided as follows:

- Lines 198-200 and 203-204: repetitions

→We thank the Reviewer for pointing this out. We have revised the sentences (Lines 200).

- Lines 231-234: number of patients already mentioned previously

→We thank the Reviewer for pointing this out. We have removed the repetition.

- paragraphs 246-254: data from the table, and please note that it is not necessary to point out the difference between OA and NOA. The OA and NOA diagnosis has already been made previously, so please avoid lines 246-248.

→We thank the Reviewer for pointing this out. We have removed the references to OA and NOA diagnoses.

- paragraphs 257-267: as the details for each group in Table 3, please summarize as follows: “no difference in all groups with a total success rate of...”.

→We thank the Reviewer for their kind suggestion. . We have revised the section based on your suggestions (Lines 247-252).

- lines 278-282, in the introduction

→We thank the Reviewer for their valuable advice. Although we have also described meiosis of translocated chromosomes in Lines 156-158 and 162-166, we have included this section in the Discussion to provide a more detailed explanation. We have not deleted these sentences because we considered them necessary to make this manuscript easy to understand by general reproductive medicine practitioners who are not experts in cytogenetics (Lines 264-268).

- line 347, , Not “surprisingly”, “as expected”

→We thank the Reviewer for pointing this out. We have corrected it (Line 333).

- lines 388-391 and 397-398 unnecessary

→We thank the Reviewer for pointing this out. We have removed the sentences.

We hope our responses adequately address the concerns raised. We have carefully revised the manuscript to address the points you have raised. We would like to thank the editor and reviewers for taking the time to review our manuscript and hope that the revised version will be approved. Once again, we thank the Editor and Reviewers very much for their comments and suggestions.

Thank you for your consideration. We look forward to hearing from you.

Best,

Tomonari Hayama, M.D.,Ph.D.

Department of GYN, Yokohama City University Medical Center

4-57 Urafunacho, Minami-ku, Yokohama City, Kanagawa Prefecture, Japan.

[Phone number] +81452615656

[Email] tommy_h@yokohama-cu.ac.jp
